# Prevalence of footrot in Swedish slaughter lambs

**DOI:** 10.1186/1751-0147-53-27

**Published:** 2011-04-14

**Authors:** Ulrika König, Ann-Kristin J Nyman, Kerstin de Verdier

**Affiliations:** 1 Swedish Animal Health Service, Kungsängens gård, SE-753 23 Uppsala, Sweden; 2 National Veterinary Institute, SE-751 89 Uppsala, Sweden

## Abstract

**Background:**

Footrot is a world-wide contagious disease in sheep and goats. It is an infection of the epidermis of the interdigital skin, and the germinal layers of the horn tissue of the feet. The first case of footrot in Swedish sheep was diagnosed in 2004. Due to difficulties in distinguishing benign footrot from early cases of virulent footrot and because there is no possibility for virulence testing of strains of *Dichelobacter nodosus *in Sweden, the diagnosis is based of the presence or absence of clinical signs of footrot in sheep flocks. Ever since the first diagnosed case the Swedish Animal Health Service has worked intensively to stop the spread of infection and control the disease at flock level. However, to continue this work effectively it is important to have knowledge about the distribution of the disease both nationally and regionally. Therefore, the aims of this study were to estimate the prevalence of footrot in Swedish lambs at abattoirs and to assess the geographical distribution of the disease.

**Methods:**

A prevalence study on footrot in Swedish lambs was performed by visual examination of 2000 feet from 500 lambs submitted from six slaughter houses. Each foot was scored according to a 0 to 5 scoring system, where feet with score ≥2 were defined as having footrot. Moreover, samples from feet with footrot were examined for *Dichelobacter nodosus *by culture and PCR.

**Results:**

The prevalence of footrot at the individual sheep level was 5.8%, and *Dichelobacter nodosus *was found by culture and PCR in 83% and 97% of the samples from feet with footrot, respectively. Some minor differences in geographical distribution of footrot were found in this study.

**Conclusions:**

In a national context, the findings indicate that footrot is fairly common in Swedish slaughter lambs, and should be regarded seriously.

## Background

Footrot is a world-wide contagious disease in sheep and goats. It is an infection of the epidermis of the interdigital skin, and the germinal layers of the horn tissue of the feet. The causative agent is *Dichelobacter nodosus (D nodosus)*, in conjunction with *Fusobacterium necrophorum *[1]. Predisposing factors are humid and warm weather conditions, and inter-digital dermatitis is a precursor to footrot. The severity of footrot depends on the strain of *D nodosus *and the environmental conditions [2]. The clinical symtoms are typical foot lesions, and lameness due to the painful lesions. Lameness is, however, not a consistent clinical symtom in all sheep affected with footrot. Footrot may vary in severity from inflammation of the interdigital skin to complete under-running of hoof horn.

The lesions can be described with a 0 to 5 scoring system [2], as shown in Table 1. Score 1 is not consistent with disease and occurs in herds with and without footrot. On a flock level, footrot can be clinically classified as virulent or benign according to e.g. the Australian system. In a flock with more than 10% of feet with score 4 lesions present at a time when footrot has had an opportunity to express itself fully, the diagnose is virulent footrot. Benign footrot on a flock level is characterised by lesions that usually not extend beyond the interdigital skin [2]. The diagnosis footrot is clinically established based on the severity of the foot lesions (score 2-5), and can be verified with presence of *D nodosus *in bacteriological tests with different specificity. *D nodosus *can be detected by Gram staining and microscopy [2], culture on hoof agar plates or by PCR [3]. By determining the protease thermostability by gelatine gel test, different strains of *D nodosus *can be defined as virulent or benign [2].

**Table 1:** Scoringsystem for footrot (2)

Score	Explanation
**0**	Healthy foot.
**1**	Slight to moderate inflammation confined to the interdigital skin.
**2**	A necrotising inflammation of the interdigital skin which involves part or all of the soft horn of the axial wall of the digit.
**3**	A necrotising inflammation with underrunning of part or all of the soft horn of the heel and the sole.
**4**	Underrunning extending to the abaxial edge of the sole of the hoof.
**5**	Necrotising inflammation of the laminae of the abaxial wall with underrunning of the hard horn of the hoof.

The first case of footrot in Swedish sheep was diagnosed in 2004 [4]. Due to difficulties in distinguishing benign footrot from early cases of virulent footrot and because there is no possibility for virulence testing of strains of *D nodosus *in Sweden, the diagnosis is based of the presence or absence of clinical signs of footrot (score ≥2) in sheep flocks. There is yet no Swedish system to differentiate benign from virulent footrot on a flocklevel and since the Swedish sheep breeds and weather conditions are different from the Australian it is not wise to simply adapt the Australian system. Ever since the first diagnosed case the Swedish Animal Health Service has worked intensively to stop the spread of infection and control benign and virulent footrot at flock level [5]. However, to continue this work effectively it is important to have knowledge about the distribution of the disease both nationally and regionally. Therefore, the aims of this study were to investigate the prevalence of footrot in Swedish lambs at abattoirs and to assess the geographical distribution of the disease. The Swedish sheep population consists of approximately 220 000 ewes in 10 000 flocks, geographically predominating in the southern part of the country. The average flock size is about 30 ewes and the most common breed is "Gotland pelt sheep" (Sheep Farmers Union: personal communication).

## Methods

In cooperation with the National Veterinary Institute (NVI), the Swedish Animal Health Service conducted a prevalence study on footrot in slaughter lambs in Sweden in the autumn of 2009. The study was conducted in early September, close to the summer which is the season in Sweden when weather conditions (relatively high temperature and humidity) most easily can provoke the development of footrot.

The selection of slaughterhouses was based on the number of lambs slaughtered per slaughter house in 2008 and the geographical location of the slaughter house. Six (out of totally 44) slaughter houses throughout the country were selected to participate. These slaughter houses covered 65% of the total number of slaughtered lambs in Sweden during the most intense slaughter period (August to October) in 2008.

The numbers of slaughter lambs in Sweden are large enough (203 000 in 2008) to assumed that the distribution of footrot in this population is normally distributed throughout the country, and a random sample from this population will give a representative prevalence of footrot in Swedish slaughter lambs. The sample size was calculated using the sample size equation **n = (Z**^**2 **^**× P (1 - P))/d**^**2**^. The expected prevalence (P) was set to 50% since no previous prevalence of footrot of Swedish slaughter lambs was available, and the precision (d) in our prevalence was set to 5%. Using this equation n equals to approximately 400 lambs. Given that we do not detect *D nodosus *in 25% of the footroot cases with score ≥2 when using bacteriology, the total sample size of slaughter lambs was set to be 500. The number of feet to collect from each slaughter house was determined by the proportion of lambs slaughtered in 2008 (Table 2).

**Table 2:** Descriptive data concerning total number of slaughter, sampled and footrot positive lambs from a footrot prevalence study preformed in Sweden investigating slaughtered lambs from six slaughter houses

Slaughter house	Number of slaughtered lambs in Sept. 2008	Total number of lambs sampled during the study	Number of lambs sampled per day under study period	Number of lambs with one or more feets diagnosed with footrot
**Linköping**	6439	200	20	6
**Skara**	4131	128	13	7
**Visby**	3359	104	11	8
**Hörby**	1240	38	4	3
**Krylbo**	476	15	1-2	3
**Norrbottensgården**	474	15	1-2	2

**Total**	**16 119**	**500**		**29**

To assess if there were any differences in prevalence of footrot in different areas of Sweden the results of the slaughter houses was amalgamated according to geographical areas; southern parts of Sweden (three of the slaughter houses representing 366 slaughtered lambs), Gotland (an isolated island of Sweden; one slaughter house representing 104 slaughtered lambs) and northern parts of Sweden (two slaughter houses representing 30 slaughtered lambs). Differences in prevalence between the geographical areas were then assessed by statistic analysis using univariable logistic regression analysis, where the lamb was the study unit and the dependent variable was footrot status of the lamb (absent or present, according to previously described definition). A *P*-value of ≤0.05 was considered statistical significant. The statistical analyses were done using Stata Software (StataCorp., 2009; Stata Statistical Software: Release 11.0; College Station, TX, USA: StataCorp LP.).

Feet were collected from August 31 to September 17. The collection was done by the staff at the slaughter houses. All four feet were cut off from every tenth slaughtered lamb (spread evenly over each day as an effort to not sample lambs from same farms), put into plastic bags, labelled with date and submitted in a padded envelope by regular mail to the NVI. On Monday-Thursday slaughter, feet arrived to the laboratory at the NVI within 24 hours. On Friday slaughter, feet were stored in refrigerator over the weekend and thus arrived after four days (Tuesday morning). Information about identity of lamb and sheep farmer was not possible to collect, because of practical reasons.

**Figure 1:**
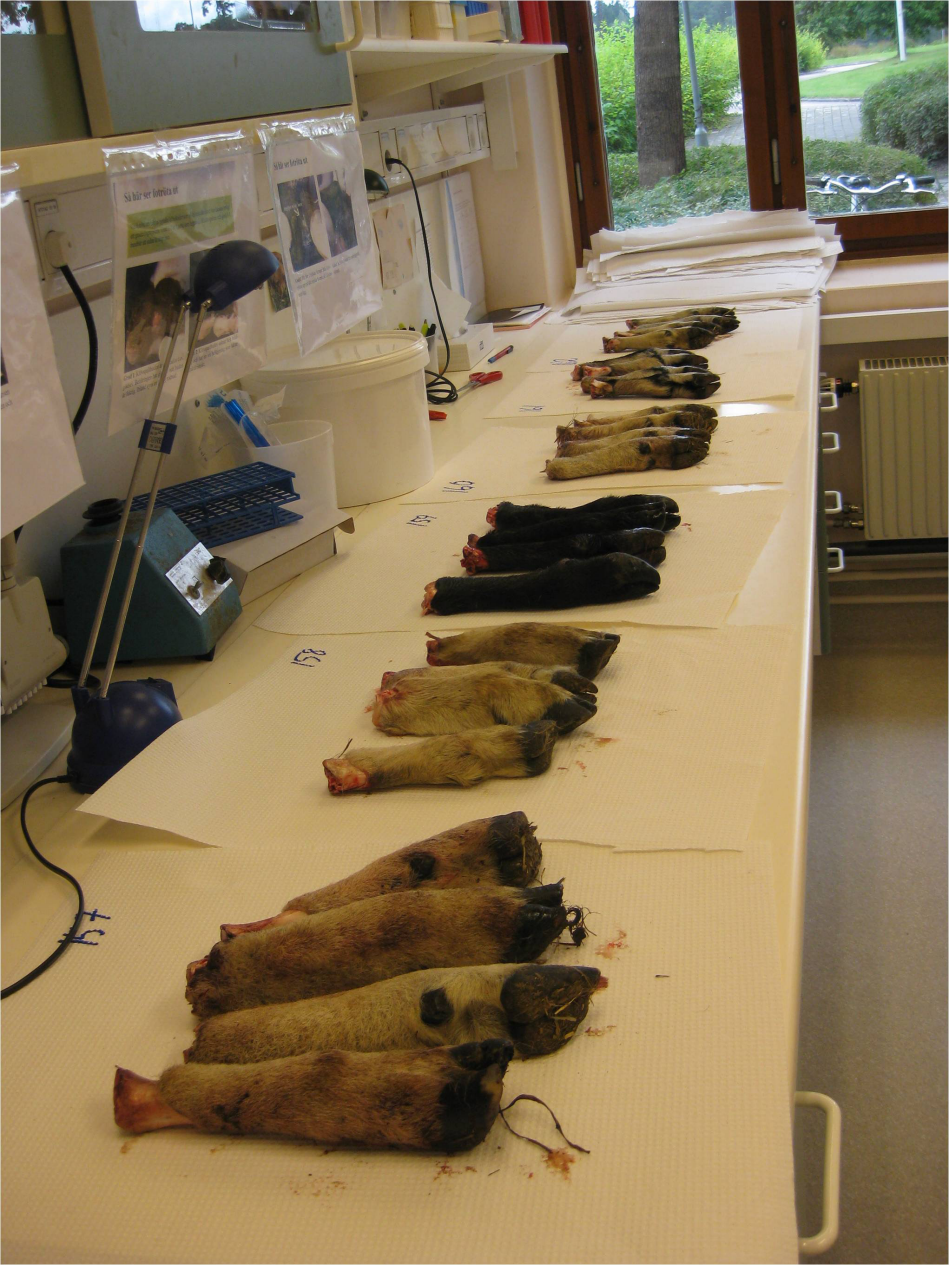
**Lamb feet for visual inspection**.

At the laboratory, the feet were inspected visually (figure 1) by at least one (and often by four) technicians, specially trained for the purpose. The Australian scoring system [2] was used for the assessment. Score 0 to 1 was regarded as healthy feet. Score 2-5 lesions were defined as footrot. Other foot lesions were additionally registered from the second day of the study (September 2).

Bacteriological samples were collected with a sterile wooden stick (8) from the interdigital skin of feet with footrot. The samples were placed in Amies transport medium with charcoal and on the same day streaked on hoof agar plates for bacteriological analysis [5]. The plates were incubated at 37°C under anaerobic conditions for 4-6 days. Colonies with characteristic appearance were identified by Gram stain [5] and real-time PCR (NVI in house PCR). In addition to culturing on agar, sampling with "ESwab" (Copan Innovation Ltd, Brescia, Italy) for real-time PCR was performed. Samples for culture and PCR were collected from only one foot from each lamb, regardless of whether footrot was present on one or more feet.

## Results

### Prevalence of footrot

**Figure 2:**
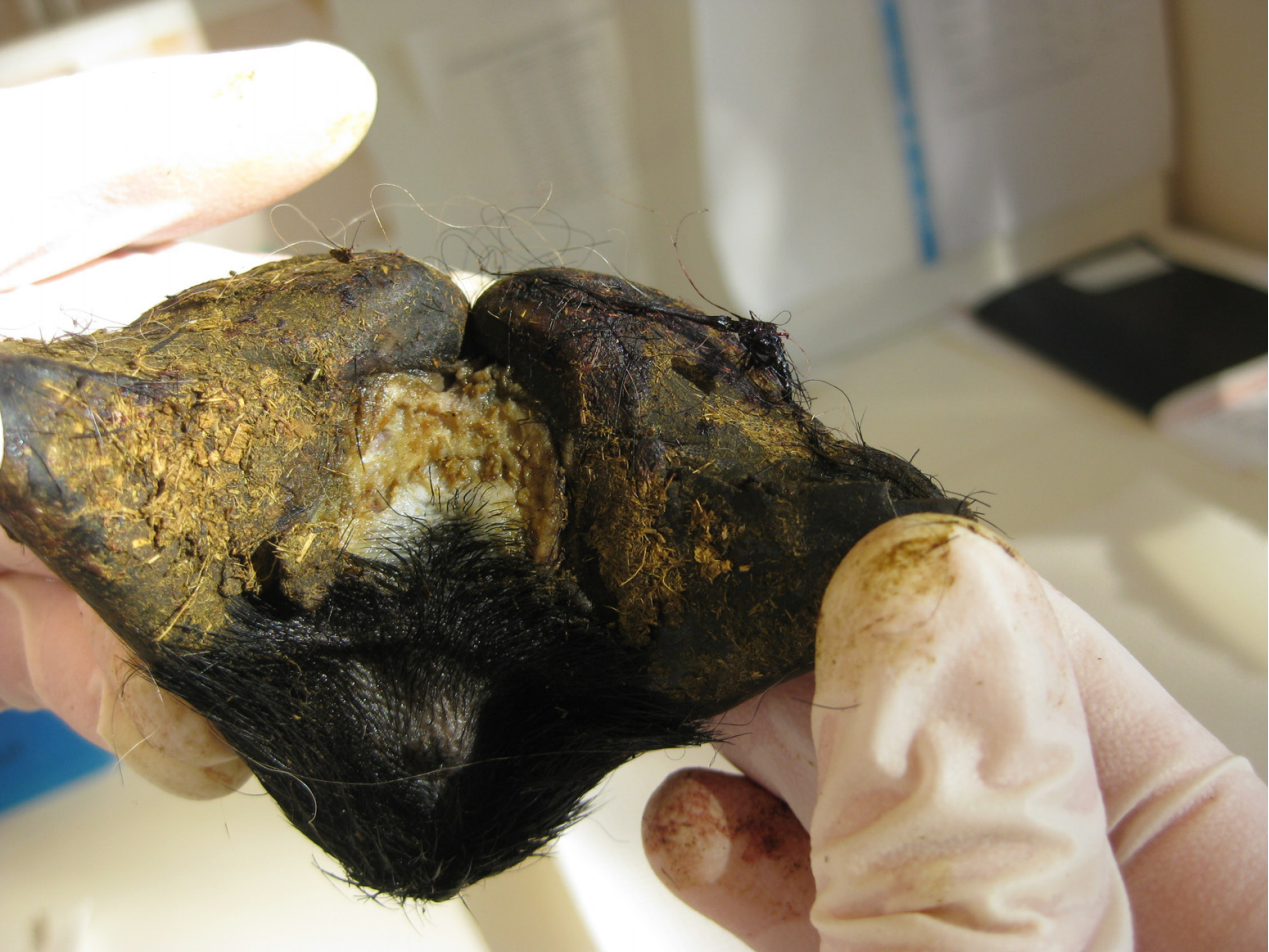
**Footrot score 2 in a cut off foot**. The lesion is visible between the claws, in the middle of the picture.

A total of 2000 feet from 500 slaughtered lambs was investigated, and 60 feet with footrot score 2 or more were found (table 3, figure 2). These 60 feet originated from 29 different lambs. Of these, 12 lambs had one foot affected, seven lambs had two feet affected, six lambs had three feet affected and four lambs had all feet affected with footrot. The prevalence of footrot at the individual level was thus 5.8% (29/500, 95% confidence interval (CI): 3.9% - 8.2%).

**Table 3:** Distribution of footrot lesions in slaughter lambs collected at six slaughterhouses participating in a footrot prevalence study performed in Sweden (2009)

	Score 0-1	Score 2	Score 3	Score 4	Total
**Number of feet**	1940	57	1	2	**2000**
**Number of lambs**	471	27	1	1	**500**

### Geographical distribution

**Figure 3:**
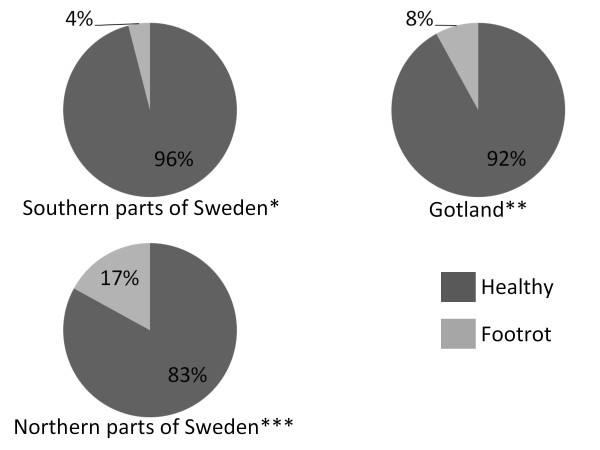
**Distribution of slaughter lambs with or without footrot in different geographical regions in Sweden**. *Slaughter houses in Hörby, Linköping and Skara; ** Visby; *** Krylbo, Norrbottengården.

The geographical distribution of lambs with footrot is presented in Figure 3. The prevalence of footrot was significantly (*P *= 0.008) higher in northern Sweden (16.7% (95% CI: 5.6% - 34.7%)) than in southern Sweden (4.4% (95% CI: 2.5% - 7.0%)). There was no significant difference between the prevalence of footrot on Gotland (7.7% (95% CI: 3.4% - 14.6%)) compared to the other regions.

### Dichelobacter nodosus

*D nodosus *was detected by real-time PCR in 28 of 29 samples (97%), and by culture in 24 of the 29 samples (83%) from feet with footrot (score 2-4).

### Other foot lesions

Separation of white line was recorded in feet from 47 lambs. Erosions were observed on feet of six lambs and hyperplasia of the interdigital skin from feet of four lambs. More than one lesion per foot and lamb were commonly observed. Maggot infestations were recorded in two feet from a lamb with footrot of score 4.

## Discussion

The study was designed to describe the prevalence of footrot in lambs at the individual level, nationally. An investigation of feet from a random sample of slaughter lambs will then be representative for the whole population of Swedish slaughter lambs, and will provide a good estimate of the prevalence of footrot. Compared with inspection and sampling in live flocks the abattoir sample collection strategy proved to be simple and effective, and ensured that samples from many lambs from a large area were examined.

There are currently no national records of prevalence of various diseases and lesions in sheep, but according to the Swedish Animal Health Service database disease and injury remarks at slaughter are rare. The most commonly recorded remarks for slaughter lambs and sheep during 2008-2009 were pneumonia (1.3 and 1.0% for lambs and sheep, respectively) and pleuritis (0.7% and 3.3% respectively). In comparison with those numbers the finding in this study of a prevalence of footrot of 5.8% can be considered as high, and together with the fact that footrot has consequences for sheep health and sheep welfare, this suggests that footrot in Sweden should be taken seriously.

A survey was conducted on 481 apparently healthy slaughter sheep in Sweden in 2004, showing presence of inter-digital dermatitis in 6.0% of the sheep, and 0.4% of the sheep had underrunning of the horn [6]. Clinical and bacteriological diagnosis of footrot was not sufficient at that time in Sweden, but possibly some of these findings were due to footrot. Our findings of 5.8% prevalence and a distribution of footrot throughout the country suggest that the disease was present in the country before the first reported case in 2004, since trading of sheep between different regions in Sweden is limited.

It is difficult to compare results from prevalence studies performed in other countries because there are large differences in methods (questionnaires, clinical examination, inspection at slaughter) and sampling (severity of footrot, number of feet assessed, age of animals). However, a flock prevalence of 15% was reported from New South Wales in Australia [7], and a flock prevalence of 96% was found in a survey in England and Wales [8].

The most common score of footrot in our study was score 2, as expected. Sheep with more severe footrot are unlikely to be sent for slaughter since they are under treatment with antibiotics and often are lame. The maggot infestation in the feet from one lamb, however, was not expected and unacceptable from animal welfare and hygienic points of view. The observations of other foot lesions in this study showed that white line separation was relatively common and this may have a clinical impact on the foot health of slaughter lambs.

The geographical differences in our study were not expected as most of the affected flocks so far have been situated in the south of Sweden (U.König, unpublished data).

Footrot has not earlier been reported from the north by farmers or veterinarians. Depiazzi (1998) has shown different clinical pictures in different parts of Australia due to environmental factors [9], and perhaps this also applies in Sweden. Moreover, as veterinary management of footrot by Swedish Animal Health Service only has been demanded for by sheep farmers in the southern parts of Sweden, this may have contributed to a better situation in this region compared to the north.

In our study, the consistency between clinical and bacteriological findings was very good. Sampling with ESwab and testing by PCR thus verifies the clinical diagnosis of *D nodosus *infection.

In a national context, our findings call for the launching of a footrot control program to prevent further spread of the infection. There is still a good chance to combat the disease cost effectively. Non-veterinary staff can be trained to perform clinical inspection of feet to detect footrot in flocks. In addition, feet from slaughter lambs are superior to photos and an excellent educational material to demonstrate footrot lesions.

Our study could easily be repeated at regular intervals to monitor the prevalence of footrot at a national level.

## Conclusions

The finding in this study of a prevalence of footrot of 5.8% can be considered as high, and suggests that footrot in Sweden should be taken seriously. The geographical differences in the study were not expected as most of the affected flocks so far have been situated in the south parts of Sweden. The consistency between clinical and bacteriological findings was good. One way to try to control the disease before the prevalence increases is to train non-veterinary staff to perform clinical inspection of feet to detect footrot and identify problem herds. For national monitoring of the prevalence of footrot, this prevalence study should be repeated at regular intervals.

## Competing interests

The authors declare that they have no competing interests.

## Authors' contributions

UK, AKN and KdV initiated and designed the study. UK instructed all participating personnel and AKN performed all statistical calculations. UK, AKN and KdV were all involved in the interpretation of results and drawing of conclusions, and have been equally active in writing this paper. All authors have read and approved the final manuscript.
